# Where do patients with MRI-confirmed single-level radiculopathy experience pain, and what is the clinical interpretability of these pain patterns? A cross-sectional diagnostic accuracy study

**DOI:** 10.1186/s12998-019-0273-8

**Published:** 2019-10-07

**Authors:** Hanne B. Albert, Jeanette Kaae Hansen, Helle Søgaard, Peter Kent

**Affiliations:** 1 The Modic Clinic, Odense, Denmark; 2 Research Department, Spine Centre of Southern Denmark, Lillebaelt Hospital, Middelfart, Denmark; 3 School of Physiotherapy and Exercise Science, Curtin University, Kent St, Bentley, Perth, WA 6102 Australia; 4 Department of Sports Science and Clinical Biomechanics, University of Southern Denmark, Odense, Denmark

**Keywords:** Disc herniation, Spinal nerve roots, Radiculopathy, Pain drawings, Dermatomes, Discrimination, Diagnostic accuracy

## Abstract

**Background:**

Clinicians nominate the distribution of leg pain as being important in diagnosing nerve root involvement. This study aimed to identify: (i) common unisegmental radicular pain patterns and whether they were dermatomal, and (ii) whether these radicular pain patterns assisted clinician discrimination of the nerve root level involved.

**Methods:**

A cross-sectional diagnostic accuracy study of adult patients with radicular leg pain at a hospital in Denmark. All patients had positive neurological signs (average 2.8 signs - hypoalgesia, diminished reflexes, muscle weakness, positive Straight Leg Raise test).

Part 1 (pain patterns) was a secondary analysis of baseline pain pattern data collected during a clinical trial. The pain charts of 93 patients with an MRI and clinically confirmed single-level disc herniation with nerve root compression were digitised and layered to form a composite picture of the radicular patterns for the L5 and S1 nerve roots, which were then compared to published dermatomes.

In Part 2 (clinical utility) we prospectively measured the discriminative ability of the identified pain patterns. The accuracy was calculated of three groups of six clinicians at classifying the nerve root affected in a randomized sequence of 53 patients, when not shown, briefly shown or continuously shown the composite pain patterns. In each group were two chiropractors, two medical doctors and two physiotherapists.

**Results:**

There was a wide overlap in pain patterns from compromised L5 and S1 nerve roots but some distinguishing features. These pain patterns had approximately 50 to 80% overlap with published dermatomes. Clinicians were unable to determine with any accuracy above chance whether an individual pain drawing was from a person with a compromised L5 or S1 nerve root, and use of the composite pain drawings did not improve that accuracy.

**Conclusions:**

While pain distribution may be an indication of radiculopathy, pain patterns from L5 or S1 nerve root compression only approximated those of sensory dermatomes, and level-specific knowledge about radicular pain patterns did not assist clinicians’ diagnostic accuracy of the nerve root impinged. These results indicate that, on their own, pain patterns provide very limited additional diagnostic information about which individual nerve root is affected.

## Background

Low back pain (LBP) with radiating leg pain secondary to a lumbar disc herniation (radiculopathy) is experienced by approximately 5% of all people during their lifetime [1, 2] but accounts for a disproportionate 30% of treatment expenditure for LBP [3]. Radicular pain is usually due to a combination of inflammation and ischemic compression of the nerve root [4], collectively referred to as nerve root irritation.

Appropriate treatment depends upon an accurate diagnosis. The diagnosis of radiculopathy currently depends upon a physical examination, nerve root compression signs, imaging (MRI or CT) and features within the patient history that are believed to be discriminative, such as the location and nature of the pain. In 2012 a Delphi consensus study that developed an assessment schedule for patients with low back-associated leg pain in primary care, 98% of participants rated the distribution of pain in the leg as an important contributor to the diagnosis of nerve root involvement - the highest rated item [5].

Pain radiation from the low back into the leg can be of somatic, neurogenic or visceral origin. Somatic pain originates from musculoskeletal tissue, such as a joint or muscle [6]. Neurogenic pain is a response to irritation or damage of nerve tissue and may radiate a considerable distance from the site of neural compromise. Visceral pain originates from an internal organ, such as the kidney. Somatic and visceral referred pain are believed to be diffuse, felt deep and is difficult to localise [6, 7]. In contrast, radicular pain is believed to be easier to localise, tends to be a sharp pain and may follow a pain distribution corresponding to a dermatomal pattern.

A dermatome is a cutaneous sensory area that receives its nerve supply from a specific nerve root. Charts of these sensory distributions have been made from experimental studies, however there is considerable variability in their results [8]. Muscles can also have distinctive pain patterns (myotomes), as can skeletal structures (sclerotomes) [8], due to these structures arising from different embryonic tissues.

Pain distribution may be a useful input to a cluster of signs and symptoms that diagnose radiculopathy. However, based on the notion that individual dermatomes predominantly receive their innervation from a single nerve root, there has also been a belief that radicular pain in a certain dermatomal distribution can predict the level of nerve root irritation. Clinical circumstances where precision about the nerve root level involved is particularly important include when injections or surgery are being planned, although in the case of surgery, what is observed during surgery can be the most influential reference standard. But it has long been recognised that dermatomes have wide overlap and considerable variability between individuals [9] and there is some evidence that radicular pain is often not dermatomal [10–12]. So, there are three considerations here, (i) the contribution of pain distribution to the diagnosis of radiculopathy, (ii) the contribution of pain distribution to discriminating which nerve root is affected, and (iii) the relationship between pain distribution and dermatomes.

Even if the distribution of radicular pain were not dermatomal, it is still theoretically possible that such pain patterns could be adequately stereotypical to provide some diagnostically useful discrimination between affected nerve root levels. Rankine et al. [13] showed that a stepwise discriminant analysis of pain and numbness patterns was able to distinguish between 60% of patients with L4, L5 or S1 nerve compression. Clearly, numbness and pain are not the same phenomenon and may potentially neither co-exist or co-locate. They concluded that pain patterns may have a role in predicting the compression level in patients with unisegmental radiculopathy but have limited diagnostic discrimination in the broader population of patients with back-related leg pain [13]. Therefore, we were interested in whether unisegmental L4, L5 and S1 radicular pain patterns were discriminative and whether clinicians’ discriminative ability could be improved by exposure to those pain patterns.

The aims of this study were to: (i) identify L4, L5 and S1 radicular pain patterns and judge whether they were dermatomal, and (ii) determine if these pain patterns were clinically discriminative of the nerve root level involved.

## Methods

This cross-sectional diagnostic accuracy study used two sources of data: (Part 1 – Pain patterns) a secondary analysis was performed of baseline data collected during a randomized controlled clinical trial (RCT), and (Part 2 – Clinical utility) data were prospectively collected to measure the discriminative ability of the identified pain patterns.

### Part 1 – pain patterns

#### Patient sample

Data from the single-blind RCT had been prospectively collected at the Medical Department of the Spine Centre of Southern Denmark, which is an outpatient secondary care hospital department. Detailed descriptions of the trial procedures have been published elsewhere [14]. Briefly, consecutive patients were included between November 2000 and December 2001 if they were 18–65 years of age and had all of the following: radicular pain to the knee or more distally in one or both legs, leg pain > 3 on a 1–10 point scale at first visit to the clinic, and a duration of radiculopathy between 2 weeks and 1 year. Patients were excluded if they would have been unable to participate in the rehabilitation protocol, had a spinal tumor, previous back surgery, were pregnant, or if their health status was associated with any pending litigation. Informed consent was obtained from all individual participants included in the study.

At baseline, *a medical history* was obtained and a *thorough physical examination of the spine and lower extremities,* including assessments of paraesthesia, anaesthesia, straight leg raise, reflexes and muscle testing, were undertaken for all patients by the same examiner who was blinded to treatment allocation. Patients self-completed a questionnaire pack that included anterior and posterior full-body pain drawings. *At baseline, almost all (95%, n = 172) patients had between two and four positive neurological signs (hypoalgesia, diminished reflexes, muscle weakness, positive Straight Leg Raise test), with a mean of 2.8 signs*.

Immediately following their baseline clinical examination, all participating patients also underwent an MRI examination that was obtained in an open low field 0.2 T, MRI unit using a body spine surface coil. Patients were positioned in supine with extended hips and knees, producing a slight lumbar lordosis.

The imaging protocol consisted of one localizer and four imaging sequences:

- Localizer sequence, 40/10/40 (TR/TE/flip angle), two coronal and three sagittal images in orthogonal planes, one acquisition in 32 s.
- Sagittal T1-weighted spin echo, 621/26 (TR/TE), 144 × 256 matrix, 300 mm FOV, and 11 4 mm slices, distance factor 0.20, two acquisitions in 6:01 min.
- Sagittal T2-weighted turbo spin echo, 4609/134 (TR/effective TE), 210 × 256 matrix, 300 mm FOV, and 11 4 mm slices, distance factor 0.20, two acquisitions in 8:42 min.
- Axial T1-weighted spin echo, 720/26 (TR/TE), 192 × 256 matrix, 240 mm FOV, and 15 5 mm slices, distance factor 0.25, two acquisitions in 8:49 min.
- Axial T2-weighted turbo spin echo, 6415/134 (TR/effective TE), 180 × 256 matrix, 250 mm FOV, and 15 5 mm slices, distance factor 0.25, one acquisition in 7:49 min.
- Axial images were performed on the three lower lumbar levels. If protrusions were present at higher lumbar levels, relevant supplementing axial series were performed.

The axial sequences (both T1 and T2 weighted), which are central to the diagnosis of herniations and nerve root compromise, were performed at the three lowest levels on all patients (five images each per level). If there were herniations or nerve root compromise at levels in the upper lumbar spine, additional axial sequences were performed at those levels. The slice thickness was 5 mm for axial images to compensate for the low signal-to-noise ratio due to a smaller field of view as compared to the sagittal sequences, for which 4 mm slices were used. Between 16 to 24 slices were obtained, depending on the size of the person.

All MRIs were evaluated by the same consultant radiologist, who was experienced in the use of a standardized research protocol for describing disc lesions [15] and blinded to each patient’s clinical characteristics. Intervertebral discs were classified as: normal, bulging, focal protrusion, broad-based protrusion, extrusion and sequestration, based on the American Society of Radiologists classification at that time [16, 17]. In a previous study, that included the ratings of the same consultant radiologist, *the test-retest reproducibility of localization of disc herniation using this protocol was kappa 0.72 (0.55–0.89)* [15]. The spinal level of the compressed nerve root was identified by reference to the vertebral location of the axial slice, the sagittal location of the disc lesion and the location of the nerve root relative to others at that vertebral level. Nerve root compromise was classified as; no contact, contact, displacement, compression of nerve root. *The test-retest reproducibility of identifying nerve root lesions using this protocol was kappa 0.82 (0.70–0.94)* [18].

#### Pain drawings

**Fig. 1:**
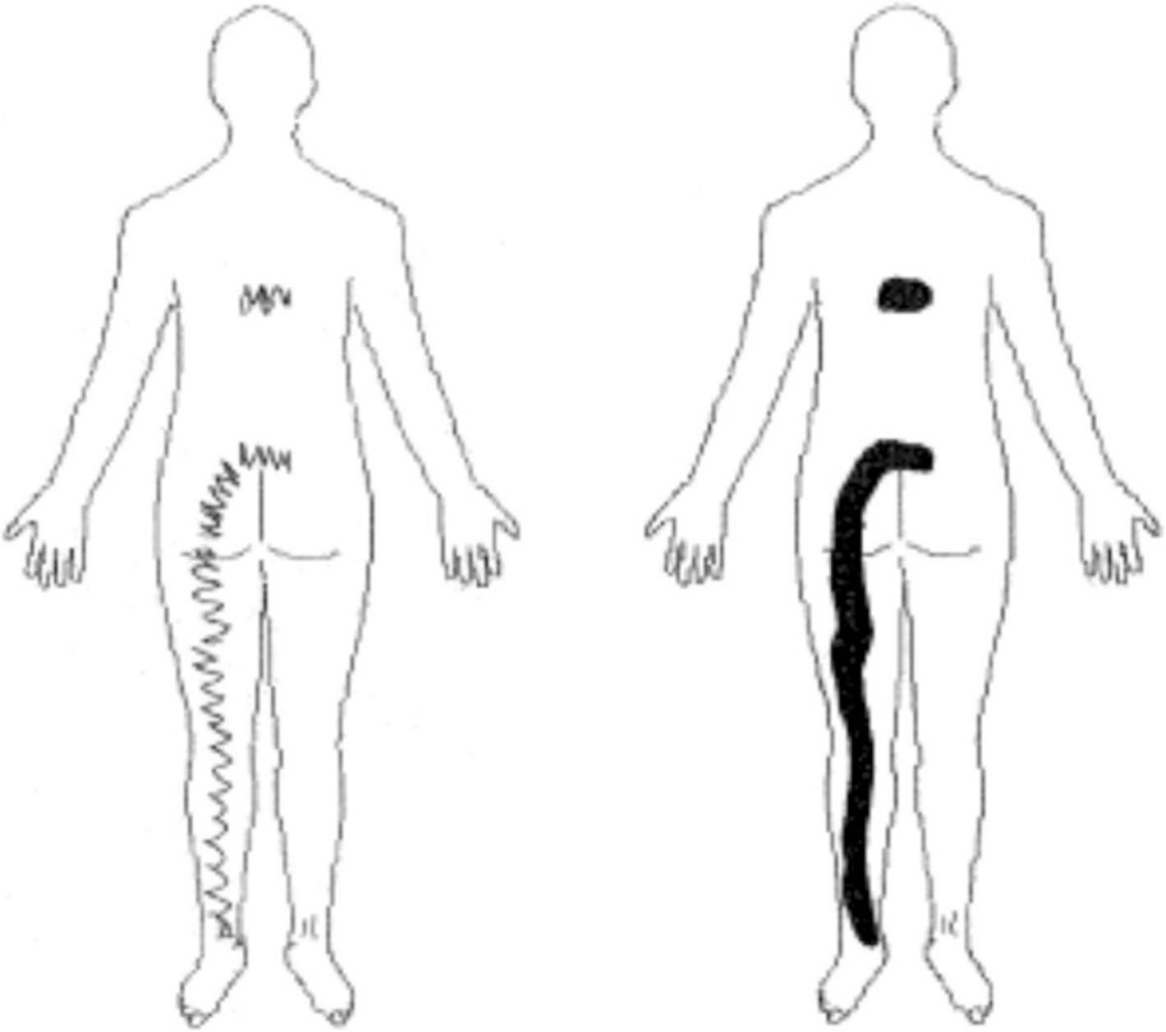
An example of one patient’s original pain drawing and after being manually delineated

Patients were asked to indicate the distribution of their pain by drawing on a pain chart. Their drawing was then discussed with the examiner to ensure comprehension, precision and that numbness and paraesthesia had not been registered as pain. As almost all patients drew with lines and/or zig-zags, prior to electronically scanning the pain chart, the total painful area was delineated by manually filling in the areas while respecting its outside boundaries (Fig. 1).

Each pain drawing was scanned into an electronic image via a standardized process: (i) all drawings were physically the same size, (ii) they were digitised using the same high-quality scanner calibrated with the same settings, (iii) any drawings indicating unilateral left sided pain were mirror-imaged so that all drawings indicated right-sided pain, (iv) all electronic images from patients with same MRI-confirmed disc-level herniation were imported into a single, multi-layered (composite) Adobe Photoshop CS3 file (Adobe Systems Incorporated, San Jose, California, USA). Each disc level-specific composite file was grey-scaled with the overall transparency of each layer calibrated so that the total density of all layers summed to 100%. Therefore, the density (darkness) of the grey scale pain areas on the composite images was representative of the proportion of patients who described pain in that area.

As it eventuated that there were only 5 patients with L4 nerve root irritation in the sample, and as we believed these to be too few to be representative, composite images were created for the L5 nerve root and S1 nerve root only. These composite images were compared with two common dermatomal images, those of Sherrington [19] and of Keegan and Garrett [20], and a subjective judgement made as to their similarity. We adapted the criteria of Murphy et al. [11], who judged a composite pain pattern to be non-dermatomal if the main commonality of the pain distribution was not contained within the area suggested by the dermatome chart to represent the cutaneous distribution of the involved nerve root. Our adaptation was to subjectively judge the proportion of the main commonality that was contained by the dermatome. As Rankine et al. [13] found that psychological distress minimally affected the utility of patients’ pain distribution for classifying the vertebral level of radiculopathy, therefore in our study, the presence of psychological distress was not an exclusion criterion, so that the sample would be more representative of the usual spectrum of patients in clinical practice.

Of the original 181 patients who participated in the RCT, the pain charts from 93 patients were included in this study. The reasons for exclusion were: multilevel disc lesions observed on MRI or L4 nerve root involvement. The pain drawing and MRI scan were obtained on the same day.

In the current study, the reference standard was the empirically-derived unisegmental radiculopathy pain distributions. This study did not seek to determine the association between the reference standard of pre-defined, commonly accepted dermatomal distributions and the presence of clinical or MRI findings, such as undertaken by Beattie et.al (2000) [10]. Instead, we took the empirical approach of ‘letting the pain drawing do the talking’ where all the pain drawings for MRI and clinically-confirmed unisegmental radiculopathy contributed to the collective image that defined pain distribution for that nerve root level. Only then did we look for an association with commonly accepted dermatomes. These two modes of inquiry address different research questions.

### Part 2 – clinical utility

In this context, we use the term ‘clinical utility’ to refer to the extent to which a test has the capacity to improve health outcomes [21, 22].

#### Clinician sample

**Fig. 2:**
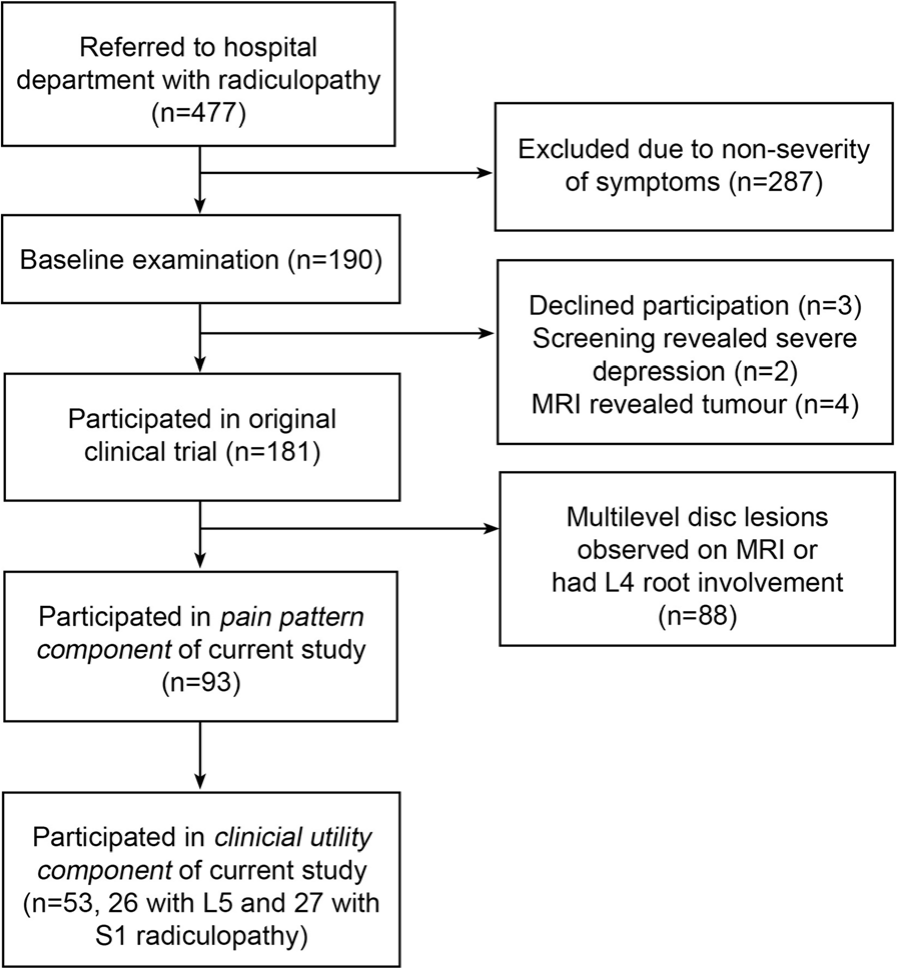
Study flow chart

Participants in this component were 18 clinicians - six physiotherapists, six medical doctors and six chiropractors – purposefully selected from a convenience sample at the Medical Department of the Spine Centre of Southern Denmark, so that two from each profession were in each of three groups. Based on our sample size estimate (see below), individuals in each group were given the task of classifying which of 53 randomly selected individual pain charts came from patients who had an L5 or S1 radiculopathy. Clinicians knew that the pain charts came from patients with either an L5 or S1 radiculopathy but were blind to all other clinical information about individual patients. This was a dichotomous choice for clinicians, as all of the randomly-selected 53 patients (26 with L5 and 27 with S1 radiculopathy) had an MRI and clinically confirmed, single-level radiculopathy involving only one of these nerve roots. A study flow chart is shown in Fig. 2.

However, the test conditions were different across the groups. Group 1 was not shown the composite pain charts and therefore *classified the pain charts based on their previous experience*. Group 2 studied the composite pain charts for 2 min and then completed the task *using a combination of their previous experience and the memory of the composite drawings*. Group 3 *could refer to the composite pain charts when classifying each individual pain chart*. This design allowed the discriminative ability of the identified pain patterns to be tested, as the groups’ knowledge of the pain patterns ranged from no knowledge through to the pain patterns being visible as they classified each patient as being likely to have either L5 or S1 radiculopathy. *Our hypothesis was that exposure to the pain patterns would increase the clinicians’ discriminative ability.* The sequence of the pain charts was randomized using Microsoft Excel (Microsoft Corp, Redmond, WA, USA). The nerve root judgements of each clinician were compared to the actual radiculopathy level for each patient and these data were double-entered in the data management system Epidata (Epidata 3.1, The EpiData Association, Odense, Denmark) by two research secretaries.

#### Sample size calculation

Using Altman’s formula, we powered the study to arbitrarily detect a difference between one group’s 80% correct ratings and a second group’s 70% correct ratings, with a power of 80% [23].

The choice of powering the study to detect a difference of 10% was based on our opinion that smaller between-group differences were unlikely to be clinically important. The resultant sample size required to detect this difference was 294, so we collected 318 ratings within each of the three groups (53 ratings from 6 observers), which was 954 ratings collectively across the three groups.

#### Comparisons

The proportion of correct ratings for each clinician group was calculated and tested for significant differences across groups using Bonferroni-adjusted inferential confidence intervals [24]. The alpha level for each comparison was determined using the following calculation: ‘alpha/number of comparisons’ = (n x (n-1))/2. As there were 3 pair-wise comparisons, the alpha level for any pair-wise comparison was reset to (0.05/3) = 0.017. Inferential confidence intervals are Bonferroni-adjusted so that if no numerical overlap occurs between compared confidence bands, a difference between proportions can be concluded with 95% confidence, and these were calculated using Microsoft Excel (Microsoft Corp, Redmond, WA, USA).

The proportion of clinicians who correctly classified whether each patient’s radiculopathy was due to an L5 or S1 nerve root irritation was calculated, as were the sensitivity, specificity and likelihood ratios of their judgements. A potential difference in clinicians’ classification accuracy between nerve root levels was examined using a Mann-Whitney-U Test (IBM SPSS v19, Armonk, NY, USA). Every MRI was coded and clinicians were making a dichotomous choice between nerve root levels (L5 or S1). Any cases with missing responses were dropped from the analysis.

## Results

### Sample

The included patient cohort (*n* = 93) had a mean age of 43.6 (SD 9.74) years and 45.2% were female. The median category of episode duration for their radiculopathy was 0.5 to 3 months, the median of their low back pain intensity score was 6.0 (IQR2.0–7.0) on a 0–10 scale, and was 4.0 (IQR 2.0–7.0) for their leg pain.

The distribution of vertebral level radiculopathy in the sample was 39 patients (41.9%) with L4/5 disc herniation and L5 nerve root irritation. Their nerve root compromise was classified as; no contact (*n* = 3), contact (*n* = 11), displacement (*n* = 19), compression of nerve root (*n* = 6).

There were 54 patients (58.1%) with L4/5 disc herniation and S1 nerve root irritation. Their nerve root compromise was classified as; no contact (*n* = 5), contact (*n* = 13), displacement (*n* = 21), compression of nerve root (*n* = 15). An overall visual characteristic of their radicular pain was that in 80.6% of the 93 patients, the pain was in a continuous band radiating into the leg.

### Pain patterns

#### L5 nerve root

**Fig. 3:**
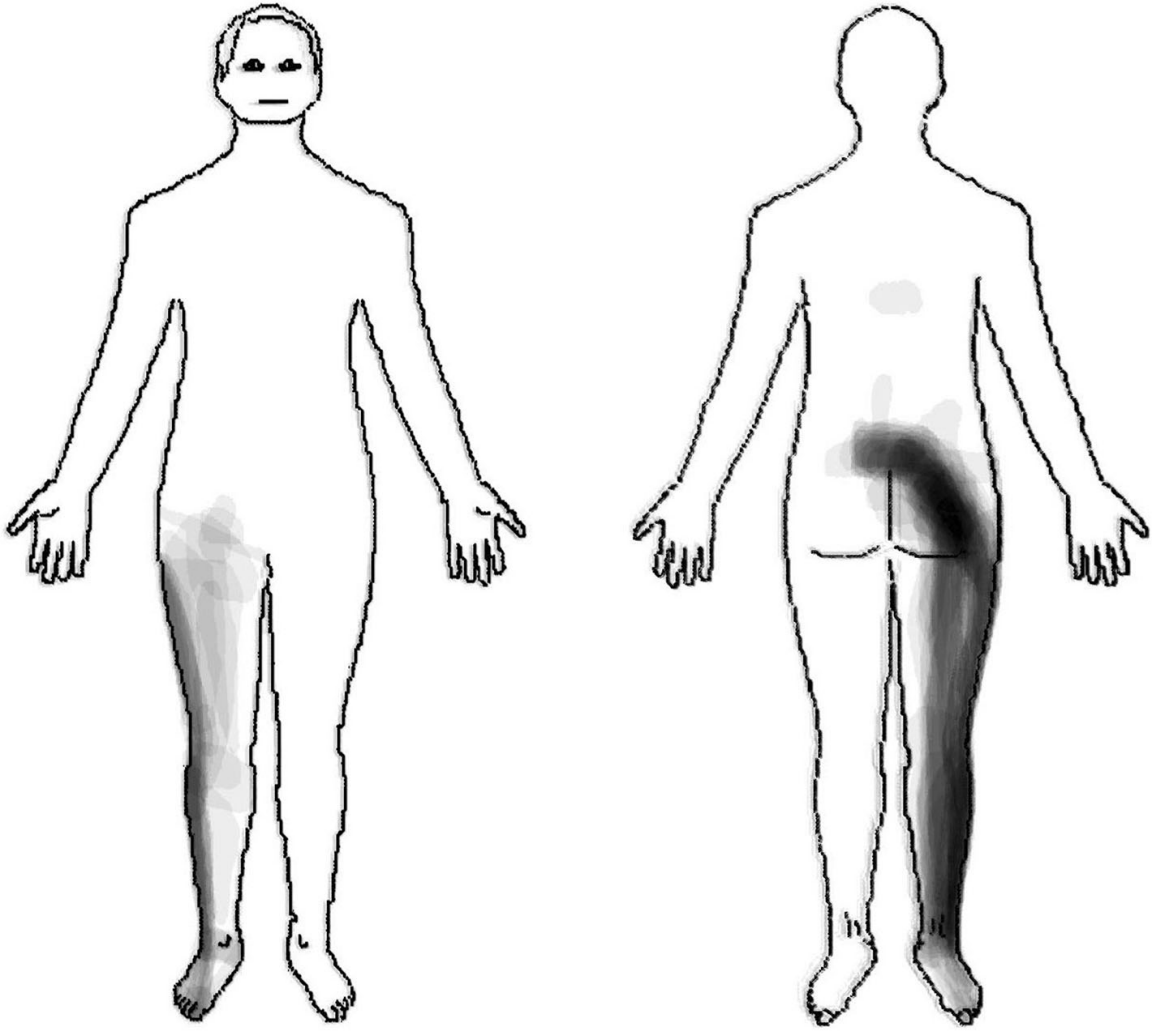
Pain pattern with L5 nerve root irritation

In these patients (*n* = 39), the pain distribution of L5 nerve root irritation commonly radiated in a longitudinal band from the centre of the lumbar area, diagonally across the central gluteal region, postero-laterally down along the thigh and calf to the ankle. In addition, pain was experienced antero-laterally along the thigh and calf to the dorsum of the foot (Fig. 3).

#### S1 nerve root

**Fig. 4:**
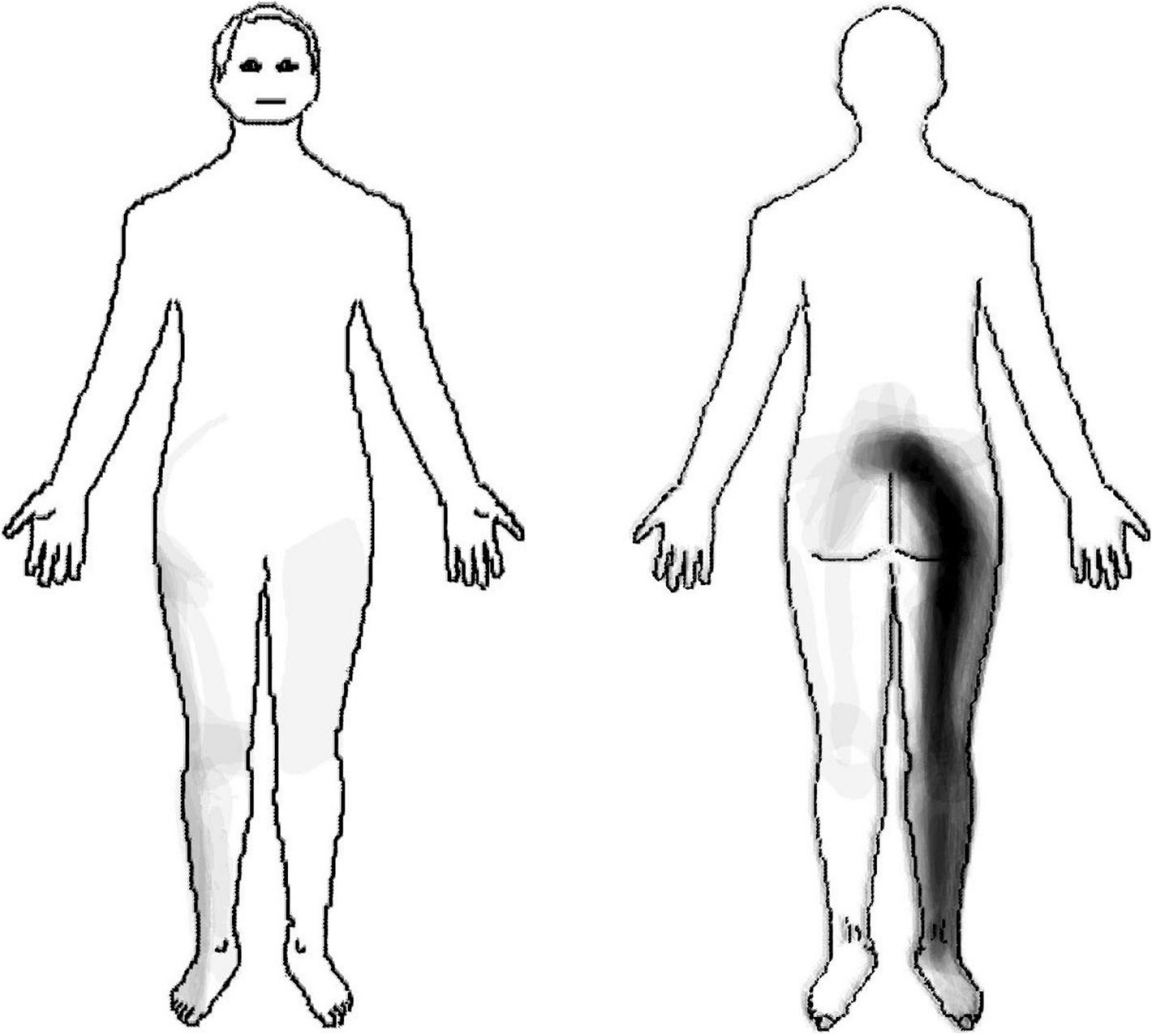
Pain pattern with S1 nerve root irritation

Patients with an S1 nerve root irritation (*n* = 54) reported pain radiating in a longitudinal band from the centre of the lumbar area, diagonally across the central gluteal region, posteriorly down along the thigh and calf to the ankle (Fig. 4).

Our subjective judgement was that approximately 50% of the main commonality in the L5 pain pattern was contained by the L5 dermatomes described both by Keegan and Garrett [20], and by Sherrington [19]. Approximately 60% of the main commonality in the S1 pain pattern was contained by the Keegan and Garrett S1 dermatome [20], and approximately 80% by Sherrington S1 dermatome [19].

### Clinical utility

The 18 clinicians who participated in this component of the project had been practising clinically for a mean of 13.9 years (SD 7.8) and been working as a clinician assessing back pain for a mean of 8.1 years (SD 5.9). Exactly half (50.0%, 9) were female.

The proportion of pain charts that were correctly classified by clinicians as being either from a patient with an L5 or S1 nerve root irritation was approximately that expected by chance (54.4 to 54.8%). The proportion correctly classified by each individual clinician ranged from 41.5 to 64.2%. There was no statistical difference across the three groups as they varied by less that 1% in classification accuracy, indicating that exposure to the composite pain drawings had no effect on clinicians’ classification accuracy (Table 1). At a whole group level, the sensitivity for judgements about the L5 root level was 55.6% (95%CI 51.0 to 60.0%), specificity 46.3% (41.9 to 50.8%), positive likelihood ratio 1.04 (0.92 to 1.16) and negative likelihood ratio 0.96 (0.84 to 1.10). At the S1 level these were sensitivity 53.7% (49.2 to 58.1%), specificity 44.4% (40.0 to 49.0%), positive likelihood ratio 1.16 (1.03 to 1.31) and negative likelihood ratio 0.85 (0.74 to 0.97). The amount of missingness in the clinicians’ ratings was (11/954 =) 1.2%. The contingency table data for the L5 root level were 260 true positives, 255 false positives, 208 false negatives, 220 true negatives and for the S1 level were 255, 260, 200, 280 respectively.

**Table 1:** Proportion of pain charts (*n* = 53) correctly classified by clinicians as being either from a patient with an L5 or S1 nerve root irritation

	Discipline	% correctly classified (n)	Group mean (95%CI)^a^
Did not see composite pain drawings	Chiropractor	64.2% (34)	54.4% (47.8 to 61.0%)
	Chiropractor	54.7% (29)	
	Medical Doctor	41.5% (22)	
	Medical Doctor	47.2% (25)	
	Physiotherapist	60.4% (32)	
	Physiotherapist	58.5% (31)	
Observed composite pain drawings for 2 min before rating patient’s pain drawings	Chiropractor	49.1% (26)	54.8% (48.1 to 61.3%)
	Chiropractor	41.5% (22)	
	Medical Doctor	64.2% (34)	
	Medical Doctor	58.8% (31)	
	Physiotherapist	56.6% (30)	
	Physiotherapist	58.5% (31)	
Could refer to composite pain drawings at any time	Chiropractor	54.7% (29)	54.8% (48.0 to 61.4%)
	Chiropractor	41.5% (22)	
	Medical Doctor	56.6% (30)	
	Medical Doctor	56.8% (30)	
	Physiotherapist	62.3% (33)	
	Physiotherapist	56.6% (30)	

^a^ The width of the confidence intervals have been adjusted (Tryon, 2001) such that when no numerical overlap occurs between confidence intervals for any particular comparison, a difference between these proportions can be concluded with 95% confidence

For each of these 53 patients, the number of clinicians who correctly classified the nerve root level of their radiculopathy ranged from 1 to 17, out of an available maximum of 18 clinicians. On average, the number of clinicians who correctly rated each patient’s vertebral level was 9.7 (SD 5.0). There was no evidence (*p* > 0.60) that clinicians’ classification accuracy varied across the two nerve root levels. Classification accuracy ranged from 6 to 94% for individual patients and this variability probably reflects how stereotypical the pain pattern was of each individual patient.

## Discussion

The first aim of this study was to identify L4, L5 and S1 radicular pain patterns and judge whether they were dermatomal. Stereotypical pain patterns for L5 and S1 radiculopathies were identified and found to only approximate dermatomal patterns, especially for L5 radicular pain. There were too few patients with L4 radiculopathy for a stereotypical pain pattern to be identified. The second aim of this study was to identify if these radicular pain patterns were clinically discriminative of the nerve root level involved, and no evidence above chance was found that they were.

### Pain patterns

Our finding that the composite pain distributions only approximated dermatomes is similar to that of Murphy et al. [11] where only a third of their 169 patients displayed pain contained within the appropriate dermatome. Our findings also are similar to those of Taylor et al. [12] who investigated radicular pain and reported that 1% or less of their 181 patients’ pain patterns overlapped 50% or more with the appropriate dermatome, and that between only 4 and 77% had *any* overlap of their pain pattern and the appropriate dermatome. There could be several reasons for this non-concordance of pain and dermatomes. It may be that the pain distribution is not only dermatomal but a combination of dermatomal, myotomal and/or sclerotomal. It could also be that individual variability is so great that dermatomal distributions can only be an approximation and this may explain the variability between published dermatomes. Central reorganization (deafferentation-induced neuroplasticity) of somatotopic maps may also play a role in dermatomal map stability and ambiguity due to pain-induced plasticity of the sensory representation of the body [25]. For example, studies of people with persistent LBP have shown large (up to 2.5 cm) shifts of the somatotopic representation in their primary somatotopic cortex [26, 27].

The current study did not seek to estimate the extent to which pain distribution contributes to the cluster of symptoms and signs that diagnose radiculopathy. However, both our composite pain distributions displayed a common pattern of a continuous line of pain, which reflects clinical belief and also reinforces the findings of Kuraishi et al. [28] who found in a sample of 73 patients that the prevalence of a continuous line of pain from the thigh to the leg was 45% with L4 or L5 radiculopathy and 35% with S1 radiculopathy. Despite this, there is limited evidence that this is discriminative between radiculopathy and other types of back-related leg pain, as few studies separate discogenic pain from verified radiculopathy. Vucetic et al. [29] found that the pain pattern of their patient sample with disc herniation was similar between those with and without surgical evidence of radiculopathy, suggesting it was not discriminative. Rankine et al. [13] found that the only pain distribution that differentiated those with radiculopathy from those without was an absence of pain in the anterior thigh and this was not a strong predictor (58% were correct for this dichotomous choice). This could be a focus of future research.

### Clinical utility

Exposure to the composite pain drawings did not improve beyond chance the participating clinicians’ ability to judge the nerve root involved, even when the patients they were rating were those whose pain drawings actually formed the composite drawing. The proportion correctly rated was within that achievable by flipping a coin and the very low sensitivity/specificity and weak likelihood ratios reflected that inaccuracy. The difficulty of accurate classification is likely to have been related to a combination of the variability of pain distribution within each nerve root level and the overlap between the pain distributions of adjacent nerve roots. We are not aware of previous studies that have tested clinical utility by directly assessing the discriminative ability of clinicians when using pain drawings.

Investigating individual test results in isolation may underestimate how they perform when combined with other clinical information during a diagnostic work-up and that could be perceived as a potential limitation of our study. However, the research design and the results of the current study are different from some previous approaches in that we did not attempt to determine the contribution of pain distribution to differentiating radicular pain from other forms of back-related leg pain in the general clinical LBP population. Instead, we designed this study to have a cohort with a very high probability of lumbar radiculopathy so that the unique contribution of the pain distribution to determining the level of the nerve root involvement could be identified. Furthermore, the study sample contained the most common levels (L5 and S1) of nerve root involvement [30].

A strength of this study is that all included patients had single-level, MRI and clinically confirmed, radiculopathy with hard neurological signs, which increased the precision of the findings. Also, the MRI findings of a herniated disc with clear nerve root impingement, that were a component of the screening used to include 91% (*n* = 85) of our patients, are those recommended by van der Windt et al. [31] as a meaningful definition of a positive imaging result for lumbar radiculopathy due to disc herniation in people with LBP. The remaining 9% (n = 8) of patients had clinical signs of radiculopathy and a single-level disc herniation at the appropriate level but no nerve root contact on MRI. They may have had nerve root contact if their posture in the MRI had been different or their nerve root irritation may have been due to chemical irritation secondary to the disc lesion. A further strength is that the study included investigation of the clinical utility of composite drawings.

The study also has some other potential weaknesses. As some people can have nerve root compression but no pain, it is theoretically possible that some of the included patients’ pain and neurological signs actually arose from a structure other than the compressed nerve root, such as inflammation of an adjacent nerve root or secondary to piriformis syndrome. Similarly, anatomical variation in pre-fixing and post-fixing of the nerve roots may introduce errors in identifying on MRI which nerve root is compromised within a vertebral foramen, though this occurrence is believed to be quite rare. For example, in the case series by Postacchini et al. [32] of 2123 patients who underwent lumbosacral myelography, 46 (2.2%) had anomalous lumbosacral nerve roots but only seven (0.4%) had anomalous L5 or S1 nerve roots that were compressed. Another potential weakness is that the MRIs were obtained between 2000 and 2001 using a 0.2 T MRI, whereas now the use of 3.0 T is emerging as the radiological standard for MRI field strength. We also acknowledge that no electrodiagnostic testing (nerve conduction studies) was performed in this study and such testing could potentially make a contribution to a reference standard for radiculopathy. However, the diagnostic performance of nerve conduction studies remains unclear [31, 33]. Similarly, magnetic resonance neurography using T-2 mapping is emerging as a more sensitive means of detecting nerve root lesions and radicular disorders [34, 35]. However, this promising technology is not widely available and is currently undergoing evaluation regarding its effectiveness for the general population.

## Conclusions

Composite pain patterns from patients in this sample, with L5 or S1 nerve root irritation, only approximated those of sensory dermatomes. Providing clinicians with level-specific knowledge of radicular pain patterns did not improve beyond chance their diagnostic accuracy of the involved nerve root level. On its own, pain distribution appears to provide minimal diagnostic information about the individual nerve root affected, even in this highly selected cohort with MRI-confirmed unisegmental radiculopathy and multiple neurological signs.

## Data Availability

The datasets used and/or analysed during the current study are available from the corresponding author on reasonable request.
